# Supplementary material for: Cell-specific gene networks and drivers in rheumatoid arthritis synovial tissues
Source: Front Immunol. 2024 Aug 5;15:1428773. doi: 10.3389/fimmu.2024.1428773 (PMC11330812; doi:10.3389/fimmu.2024.1428773)
Supplement: Supplementary file 1 [file DataSheet_1.pdf]

Supplementary Materials

A Cellular composition of synovial tissues

A.1 Clustering Synovial tissues according to cell-type enrichment scores

We collected a bulk RNA-Seq gene study of synovial biopsies (GSE89408) [26, 105], containing the gene expression profiles of 28 healthy, 152 RA and 22 osteoarthritis (OA) patients over ∼25k genes. We selected 13 cells that were previously reported in synovial tissues [15, 16, 14] and leveraged xCell [36] to infer cell-type enrichment scores from gene expression profile of each tissue in the data (Figure S1A). Then, we clustered the tissues in two clusters with hierarchical clustering, by defining the distance between patients and the Euclidean distance between their normalized xCell enrichment scores (Figure S1B).

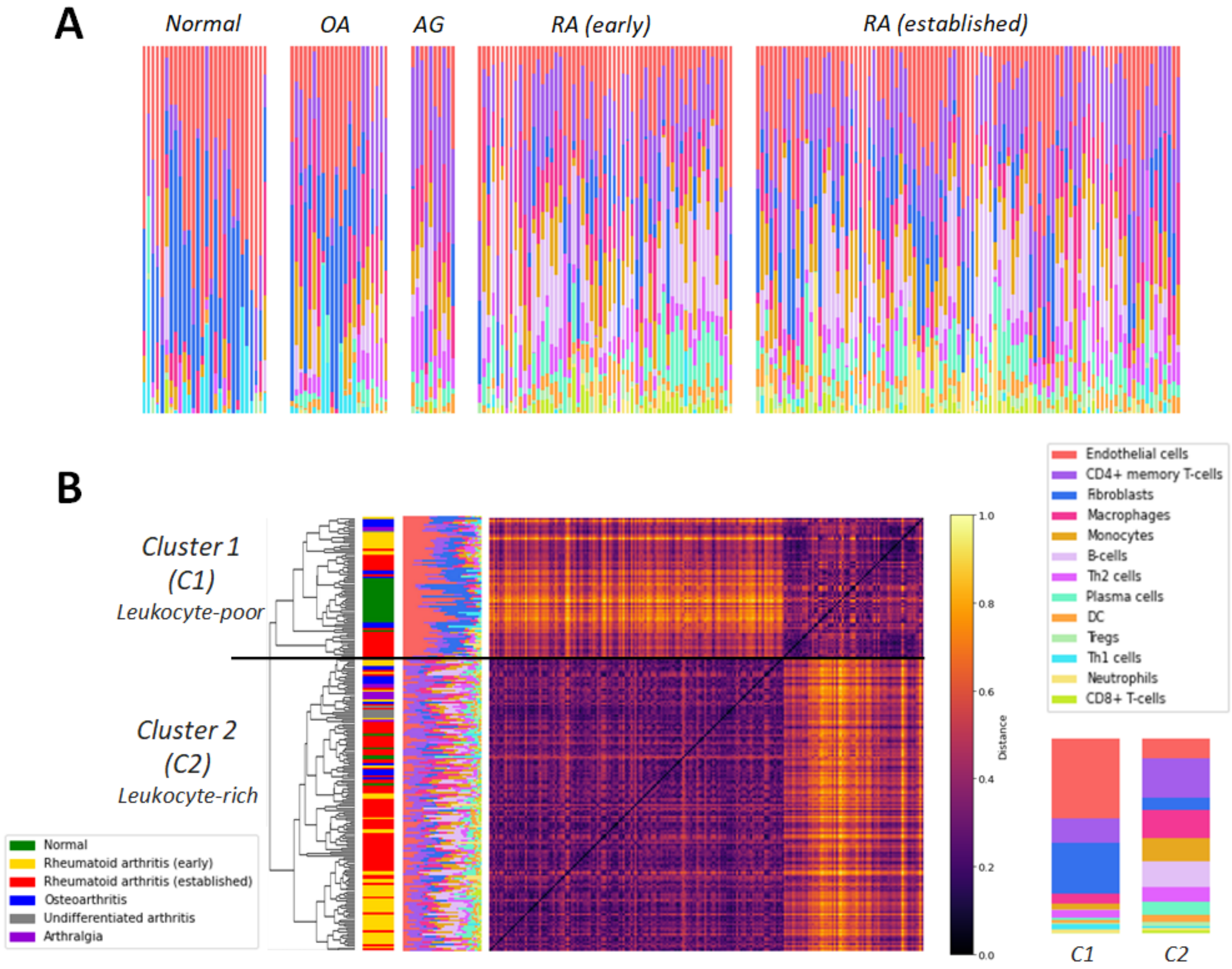

Figure S1: Cellular composition of synovial tissues. (A) The enrichment scores of 13 selected cell types are normalized and displayed in a bar plot for each sample. The samples are grouped according to their pathology: Normal, Osteoarthritis (OA), Arthralgia (AG), early RA and late RA. (B) Heatmap of the normalized Euclidean distance matrix between all synovial tissues, ordered according to their clusters from hierarchical clustering. The mean cellular composition of the two clusters (C1 and C2) is depicted on the right. Note that here we show only 13 cell types for visualization purpose.

We obtain one cluster (C1), mainly characterized by endothelial cells and fibroblasts, while the

other one (C2) contains a collection of immune related cell-types. We thus refer to these clusters as Leukocyte-poor and Leukocyte-rich, respectively. While all healthy tissues were clustered in the Leukocyte-poor cluster, a subset of early and established RA tissues were also classified in C1. Zhang & al. [15] observed a similar pattern in their study and they discuss this observation as a potential source of heterogeneity in the effectivity of RA treatments. Interestingly, they find a positive correlation between the number of leukocyte and the level of inflammation in the tissue.

A.2 Deconvolution with CIBERSORT

In the main text, we tested for differential enrichment scores across RA and control synovial tissues with xCell [36], which provided an *enrichment score* for each tested cell-types. As deconvolution methods for cellular composition are typically prone to error [115], we verified if our results were consistent with other existing methods. An extensively used pipeline for this task is CIBERSORT [40], which deconvolute directly the cellular composition of the tissues from a *signature matrix* comprised of barcode genes that are enriched in each cell-type of interest (Figure S2). To run CIBERSORT, We used the web-tool CIBERSORx (`https://cibersortx.stanford.edu/runcibersortx.php`) with a pre-loaded signature matrix comprising 22 immune cells.

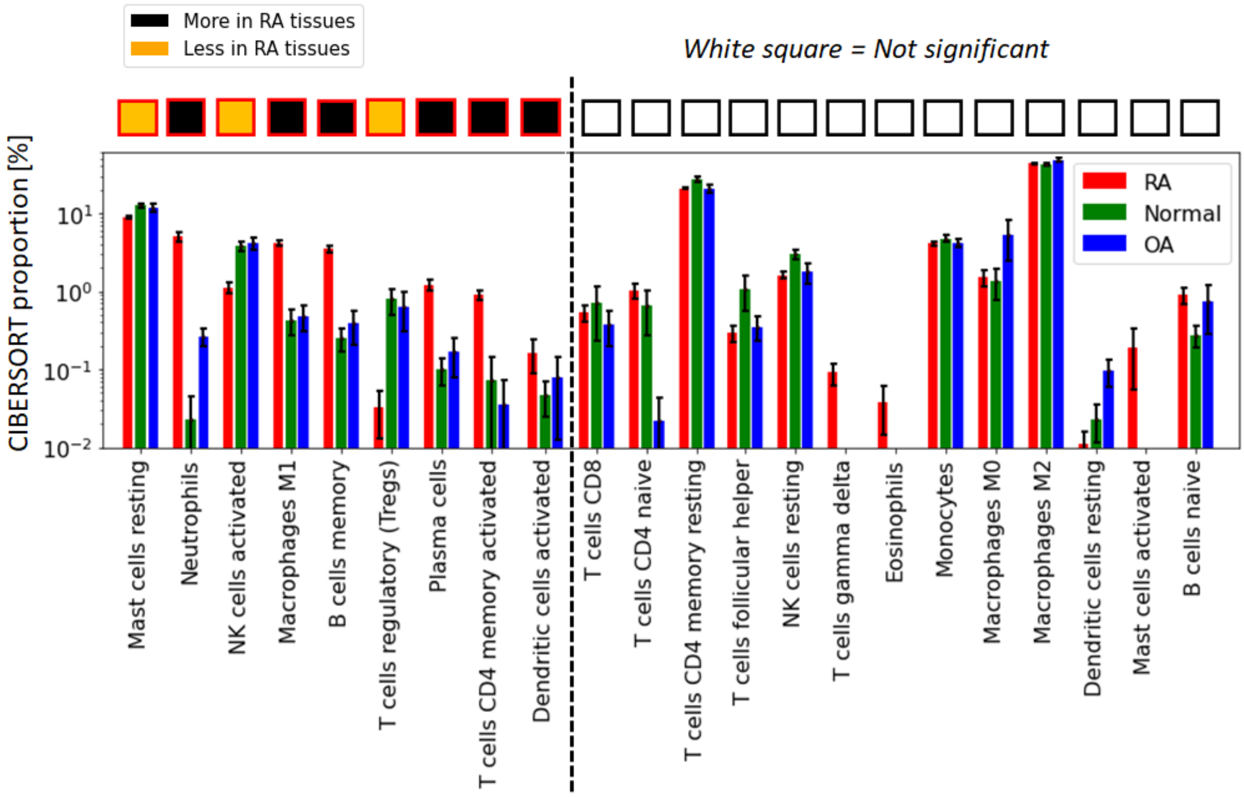

Figure S2: CIBERSORT inferred cellular composition in synovial tissues. cell-type with significantly different composition ($p < 0.05$, t-test) in both RA vs normal and RA vs OA tissues are shown on the left. Error bars are 95% confidence intervals defined as $std/\sqrt{N}$.

Overall, we find a good agreement between CIBERSORT and xCell results (Table **??**). The output agrees on Plasma cells, memory B cells, CD4+ memory T cells, and Dentritic cells. On the other hand, a few cells with significant differential enrichment with xCell were not in CIBERSORT (Eosinophils, Monocytes). Note that we could not compare other cell-types in the xCell tool, as the singular matrix for non-immune related cell-types were not available in the CIBERSORTx webtool. A complete comparison would require the construction of a signature matrix comprising all cell-types.

Table S1: Agreement between xCell and CIBERSORT cellular deconvolution on relevant cell types. Here we only show cell types that (i) could be evaluated by both tools and (2) that were overrepresented (UP) or underrepresented (DOWN) in RA synovial tissues against both OA and healthy synovial tissues with xCell enrichment scores. NS indicates non significant.

Cell Type	xCell	CIBERSORT
Memory B cells	UP	UP
Plasma cells	UP	UP
CD4+ memory T cells	UP	UP
Dentritic cells	UP	UP
Monocytes	UP	NS
Eosinophils	DOWN	NS

A.3 Explained variance of the RNA expression

A linear regressor (with coefficients denoted as β) was fitted to predict the gene expression values from the normalized enrichment scores from xCell ($c_i,\ i \in \mathit{cell\text{-}type}$), and the explained variance was then quantified as the R-Squared ($R2$) score between the predicted and original gene expression ($Y^{predict}$ and Y, respectively).

For each gene k in sample s, we predict the gene expression value as

$$Y_{ks}^{predict} = \left[\beta_{0,k} + \sum_{cells}^{ci} \beta_{ci,k}\, x_{ci,s} \right], \tag{6}$$

Then, we compute

$$\mathit{Explained\ variance} = R2_{\text{score}} \left(Y, Y^{predict} \right)$$

Strikingly, we found that the 18 cell-types discussed in the main text (Figure 1B) cumulatively account for the majority of the variance ($R2 = 61\%$) in the gene expression data (Figure S3). As expected, the explained variance increases as the enrichment scores of more cell-types are used to predict the gene expression values (from 16% with MSC only to 61% with the 18 cell-types).

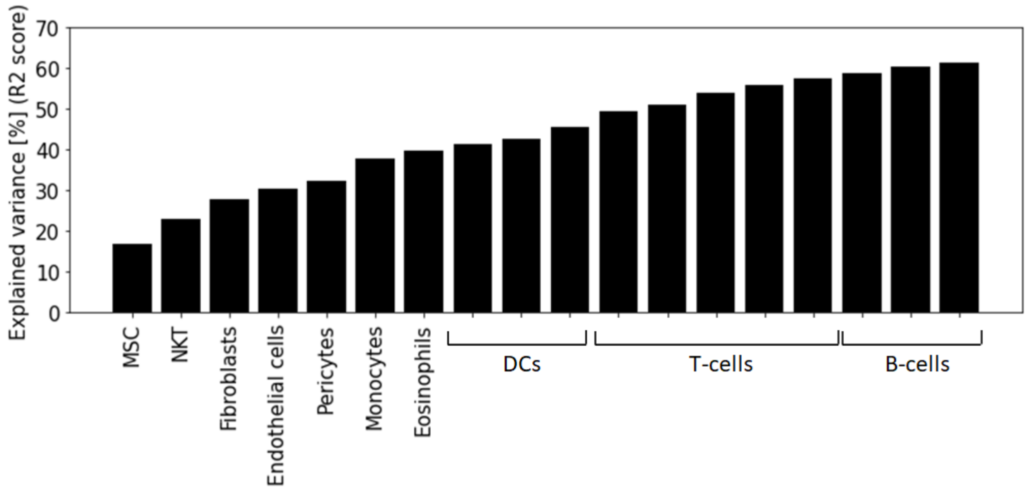

Figure S3: Cumulative explained variance of the cellular composition of the synovial tissue to gene expression. A linear regressor is fitted to predict the gene expression from each gene, where the enrichment score of the cell written in abscise is added to the previous ones (on its left) to train the linear regression.

B RA associated genes

B.1 Differentially expressed genes in synovial tissues

As discussed in the article, the variation of cellular composition in synovial tissue accounted for a large portion of gene expression variability. To capture gene expression variation caused by a changes in actual molecular state rather than changes in cellular composition across samples, we corrected the gene expression profiles to account for the tissue's cellular composition. More specifically, we considered as confounding factors the enrichment scores of cell types differentially enriched ($p < 0.05$, Student's t-test) between both RA *vs* OA and RA *vs* normal group (18 cell types), which we corrected with a simple linear regression model (Method 2.2 of the main text). Then, we used these corrected gene expression data to perform differential gene expression analysis. Differentially expressed genes (DEGs) were defined as genes obtaining a p-value lower than 0.05 in a Student's t-test between the RA and the control group, after an FDR=0.05 correction with the Benjamini & Hochberg procedure [29]. We obtained 279 DEGs.

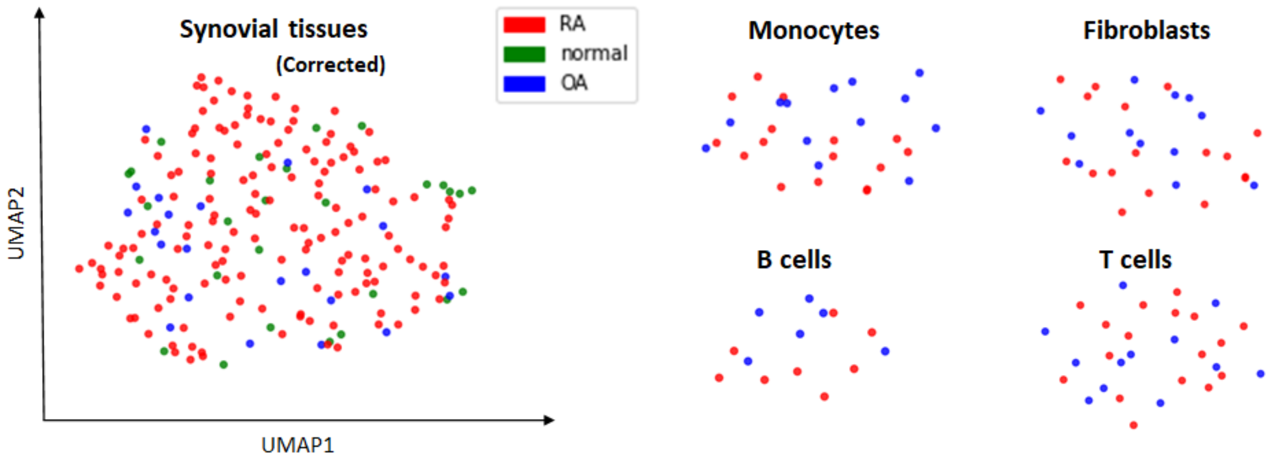

Figure S4: UMAP representation of bulk gene expression data from both cell-type specific and synovial tissues samples. The UMAP representation does not show a clear separation between RA and control groups, indicating a small difference in their gene regulation.

To complement this analysis, we also performed DEG analysis in cell-type specific gene expression

data of synovial tissues from [15]. The data consists of both bulk and single-cell gene expression data from monocytes, fibroblasts, B cells, and T cells of RA and OA synovial tissues. As the single-cell data included only 3 control patients (OA), we performed our differential expression analysis on the bulk data. Interestingly, we actually did not find any DEG for these cell types, mainly because only at most $\sim$30 samples were available for each of them. It is consistent with the gene expression data of RA and control overlapping each other in the UMAP and TSNE space (Figure S4). This observation supports the results of the first section of the main text, and confirm that there is limited gene regulatory difference between RA and control patient when the analysis is not biased by cellular composition.

B.2 Building a list of RA associated genes from databases & literature

As the DEG list we obtained in our study relies on both (i) the correct estimation of cellular composition in synovial tissues and (ii) the correction for it, it alone cannot provide a reliable list of DEGs. Thus, We combined our DEGs to several meta-analysis [58, 59, 60, 12, 13] from synovial tissues in order to obtain a robust list of DEGs. Interestingly, there were several genes differentially expressed in at least two studies. We kept the 93 genes that were found in at least 2 studies (Figure S5A).

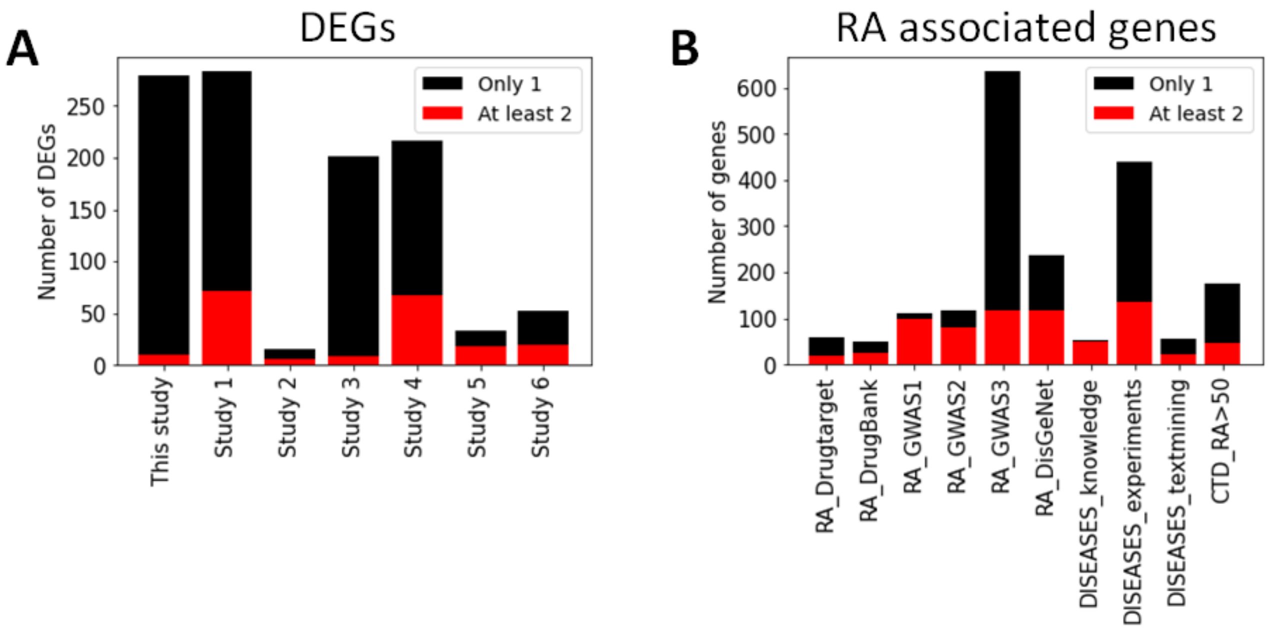

Figure S5: Number of genes across various studies & datasets involved in RA. They are colored depending on if the gene is found only in one study (black) or in at least 2 studies (red). (A) DEGs from various meta-analyses of RA synovial tissues. In addition to our own study, the list of DEG were obtained from Study1 [59], Study2 [60], Study3 [58], Study4 [12], Study5 [13] and Study6 [13] (which contains genes selected with a random forest classifier). (B) RA-associated genes from different databases. Drug_target [66], Drug_Bank [67], GWAS1 [61], GWAS2 [10], GWAS3 [62] (SNP-Disease Associations), DisGeNet [63], DISEASES_knowledge [64], DISEASES_experiments [64], DISEASES_textmining [64] and CTD [65] (kept genes with a score $>$ 50).

Likewise, we performed an extensive literature review to aggregate known genes associated with RA from different contexts. Most recent GWAS revealing genetic risk factors associated with RA were collected from studies [61, 10] and the GWASdb SNP-Disease Associations [62] database. Various genes associated with RA susceptibility were fetched from the publicly available database DISEASES [64], DisGeNet [63] and the Comparative Toxicogenomics Database (CTD) [65] databases (score $>$ 50). We also collected drug targets either already on the market or undergoing clinical trial from review [66] as well as from the DrugBank database [67] (`https://go.drugbank.com/categories/DBCAT003604`). Genes that were identified in at least two of the lists or databases were kept as the *gene literature list*, containing 259 genes (Figure S5B).

Below we provide the *Literature list* and *DEG list* that we used in our KDA analysis. Genes identified in GWAS studies [61, 10, 62] are shown in bold.

DEG_list (93 genes with 13 in GWAS):
RFX5, IRF9, TFEC, DDX60, TNFAIP6, TNFSF10, DRAM1, NMI, TNFAIP8, PLEKHO1, GZMA, IL2RG, SAMSN1, SEL1L3, LEPROTL11, ECE1, TRPV2, ENTPD1, JAK2, SLAMF8, CCL18, LY96, GZMB, TRAF3IP3, AKAP1, LPXN, PTTG1, SYK, LRMP, ANK3, CTSH, S100A8, SDC1, CECR1, AIM2, TAPBPL, GPR65, PRDX6, NKG7, COMMD8, OPN3, NAT1, NAGA, OSBPL3, CD38, IL32, DDX24, TLR7, EVI2A, GUCY1B3, MGAT4A, SEMA4D, CD8A, GALNT6, LPIN3, SLC38A6, PLCG2, QPCT, TXNDC9, IFIT1, RFTN1, TRIM21, TNS3, SRRM2, AREL1, KIAA0125, LRRC15, LCK, DNAJC15, SIRPG, FAS, GZMK, GZMH, SYNGR2, ADAMDEC1, C1GALT1C1, UCP2, FKBP11, IL21R, CXCL9, DENND1B, **RASGRP1, PSMB8, CSF1R, SMCO4, TPD52, HLA-DOB, PRKCB, ALOX5, CLIC1, MYC, IRF8, PTPRC, GSN**.

Lit_list (259 genes with 189 in GWAS):
VEGFA, SAA1, CLEC16A, IL1RN, CXCR4, IL19, IL17A, IL4, IL18, CAT, FCGRT, CCL2, HSPD1, LTA, IFNG, MECP2, CXCL10, CD14, CCL5, PIP4K2C, ALB, JAK3, TLR4, IL6, IL10, TP53, TGFB1, SPP1, IL1A, IL1B, CD19, GPX3, CYP3A4, GGT6, CCR5, NFKBIA, CCR7, CXCR3, HMOX1, IL6ST, CASP8, TAP1, CD80, IGF1, RBPJ, CCL20, PADI1, ICAM1, FAS, FLNB, PADI6, CXCL16, AGBL2, COMP, PADI3, IL15, DHODH, IL13, MMP9, SAG, VIM, **CXCR5, TNFRSF1B, EOMES, LPP, MTF1, PRDM1, ILF3, HLA-DRB1, CDK2, IQGAP1, C2, C5, TRAF1, MBL2, BCL2L15, UBASH3A, PXT1, IKZF3, CSF2, IRF8, ARAP1, MSH5, CTLA4, CCR6, C5orf30, IL11RA, TRAF6, LBH, ETS1, RASGRP1, NFKBIL1, ANKRD55, AIRE, KIAA1109, LST1, SMG7, VTCN1, CD40, ABCB1, GSDMB, HLA-DQA1, IFNGR2, CARD8, CXCL13, NOTCH4, PRRC2A, PLD4, FADS2, PTPN11, IRF5, LY6G5C, RCAN1, HLA-DPB1, CFB, HLA-DMB, LOC100506023, CCL19, FOXO1, PRMT1, IL2RB, BACH2, IRF4, CD2, CCL27, PSMB9, PTPN2, NFKBIE, CCL21, PADI4, CLIC1, LOC145837, PRKCQ, BLK, BCL3, DCLRE1C, HLA-DMA, RUNX1, HLA-DPA1, PUS10, FAM107A, BAD, SWAP70, RCOR1, NGF, BAG6, CLNK, APOM, RAD51B, TNFRSF14, UBE2L3, TAGAP, PSMB8, COG6, BAX, ZNF438, TNPO3, CALCR, ATM, GATSL3, AFF3, RELA, CD28, DDX6, IL21, IL20RA, HSPA14, FADS1, RTKN2, TNFAIP3, CD5, TXNDC11, ETV7, PAPOLG, FGFR1OP, FCGR2B, ICOS, STAT4, P2RY10, PANK4, FCGR2A, TEC, REL, HLA-C, ATG5, CDK5RAP2, CFLAR, C1QBP, PDE2A, PRKCB, PTPRC, CD83, PLCL2, ISG20, MICA, SPRED2, TYK2, TAPBP, CEP57, FADS3, ANXA3, FNDC1, IL2RA, PTPN22, IL6R, CD226, TAP2, JAZF1, TPD52, HLA-DOB, IL23R, KIF5A, ARID5B, MYC, CSF3, GATA3, TNF, SYNGR1, ANXA6, FCRL3, DPP4, AHNAK2, CSNK2B, ICOSLG, DOK6, CELF2, AIF1, LY6G6F, ACOXL, NCF2, PADI2, WDFY4, GPANK1, YDJC, MMEL1, CDK6, PPIL4, PXK, IL2, LY6G6C, SLAMF6, CD84, ZMIZ1, PRKCH, FAM167A, SH2B3, HLA-B, IRAK1, HLA-DQB1, HLA-A**.

C Shared Key driver genes (KDGs) across networks

In the main text, we identified many key driver genes (KDGs) across the tested networks with key driver analysis (KDA). We checked if these KDGs tend to be the same across networks or if on the other hand, the identified genes across networks were independent. Our results, displayed in Figure S6, indicated that a subset of KDGs were found consistently in the majority of the network. More precisely, the top 20 genes were found in 75% of the networks and more than 500 were found in more than half of the tested networks. This is much higher than what we would expect if the KDG were independent (red line in Figure S6).

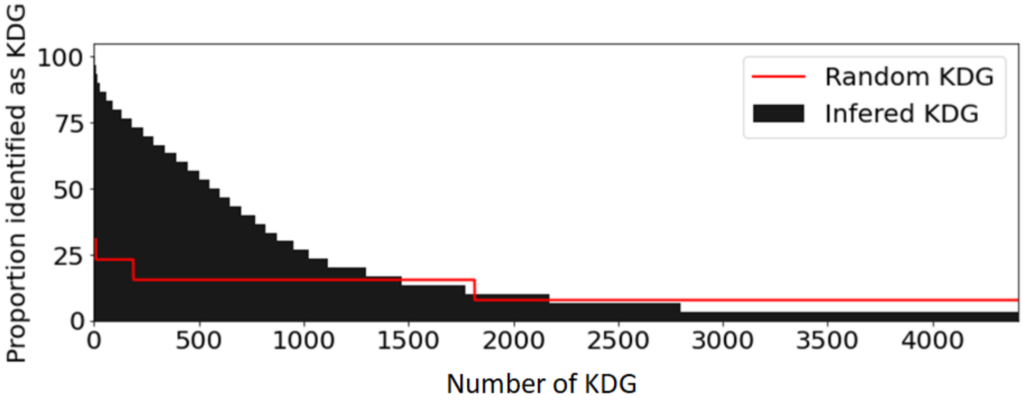

Figure S6: Gene distribution of the proportion of networks in which a given gene was found. The most commons KDGs are found in 80% of the networks while only 25% would be expected if genes were randomly assigned as KDG (red line).

Top 100 KDG list (ordered from highest to lowest score, 11 KDGs were also in GWAS [61, 10, 62] marked in bold): PTPN6, HLA-E, HLA-F, GBP1, LCP2, GLIPR1, **HLA-A**, CTSS, SRGN, CTSH, CASP1, **HLA-B**, **HLA-C**, CD44, IFI30, TNFAIP8, CCL5, **PSMB8**, TAP1, ICAM1, ARHGDIB, PSME1, B2M, THEMIS2, SP100, MYD88, **TNFAIP3**, PLAUR, ARPC1B, ITGB2, **HLA-DMA**, CYBA, HLA-G, **PSMB9**, CXCL10, IL7R, CTSC, BCL2A1, CTSB, TNFSF10, SP110, CD86, CD48, CORO1A, PSMB10, PLEKHO2, LYN, EVI2B, TPP1, IFITM2, **ISG20**, NCF4, BTN3A3, IFITM3, UBE2L6, **CFLAR**, MAN2B1, STK10, **NCF2**, WARS1, CD53, IFITM1, FAS, RGS19, LAPTM5, **PLEK**, RAC2, CD74, TNFAIP2, PECAM1, PLAAT4, **HLA-DQB1**, BIRC3, TIMP1, IL1B, NFKBIA, IRF1, STAT1, PHF11, IFIT2, COTL1, NMI, PLSCR1, OAS1, MX2, IFNGR1, CAPG, LGALS3, **NFKBIE**, HMOX1, RAB27A, RAB31, GBP2, CCL2, FYN, **TNFRSF1B**, ADGRE5, CXCR4, SERPINB1, LITAF.

D Supplementary Figures and Tables

Table S2: List of networks involved in our study. GIANT RIMBANET and PPI were downloaded from a public database while PANDA and LIONESS networks were computed specifically for this study. H refers to healthy donors.

Network(s) name	Type	Method	Used for	Number of networks
PANDA_Synovial_Tissue	Bipartite	PANDA, LIONESS*	TF reg. score	152 RA, 22 OA
PANDA_FLS	Bipartite	PANDA, LIONESS*	TF reg. score	18 RA, 12 OA
PANDA_Synovial_Monocyte	Bipartite	PANDA, LIONESS*	TF reg. score	17 RA, 13 OA
PANDA_Synovial_Bcell	Bipartite	PANDA, LIONESS*	TF reg. score	8 RA, 7 OA
PANDA_Synovial_Tcell	Bipartite	PANDA, LIONESS*	TF reg. score	17 RA, 13 OA
GIANT_DC	Bayesian	GIANT§	KDA	1
GIANT_NKT	Bayesian	GIANT§	KDA	1
GIANT_Monocyte	Bayesian	GIANT§	KDA	1
GIANT_Tcell	Bayesian	GIANT§	KDA	1
GIANT_Bcell	Bayesian	GIANT§	KDA	1
GIANT_Fibroblast	Bayesian	GIANT§	KDA	1
GIANT_Adipocyte	Bayesian	GIANT§	KDA	1
GIANT_Tonsil	Bayesian	GIANT§	KDA	1
GIANT_LN	Bayesian	GIANT§	KDA	1
GIANT_Blood	Bayesian	GIANT§	KDA	1
GIANT_Spleen	Bayesian	GIANT§	KDA	1
RIMBANET_Multitissue	Bayesian	RIMBANET†	KDA	1
PPI	Undirected	StringDB†	KDA	1

* Computed in this study from bulk RNAseq [26, 15].
§ GIANT networks downloaded from `https://hb.flatironinstitute.org/download`.
† RIMBANET and PPI downloaded from `http://mergeomics.research.idre.ucla.edu/samplefiles.php`.

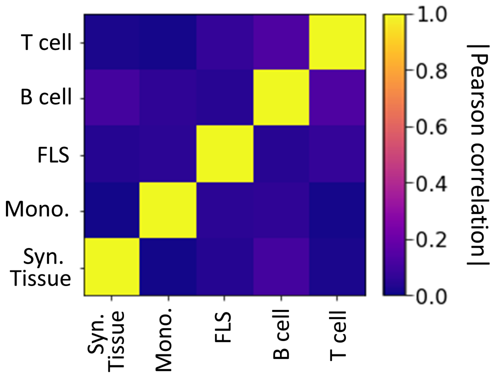

Figure S7: Heatmap of the Pearson correlation between the differential gene expression (t-score) in each tissue type (Synovial tissue, monocyte, FLS, B cell, and T cell.

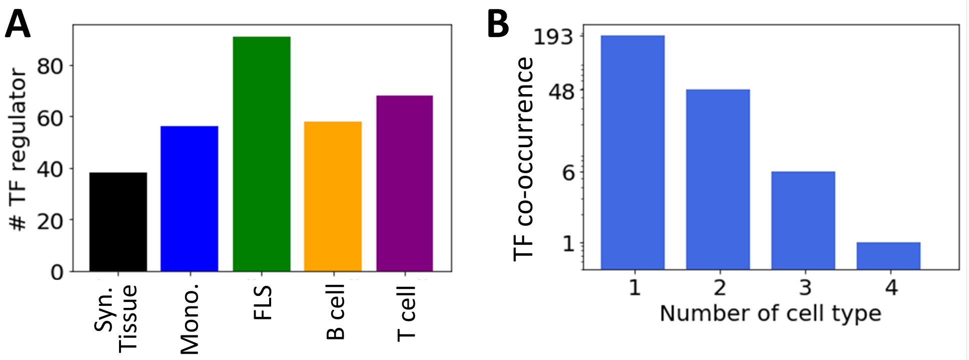

Figure S8: (A) Number of key regulator TFs identified in each cell type. (B) Number of cell type TFs regulators are identified into (note the log-scale on the y-axis).

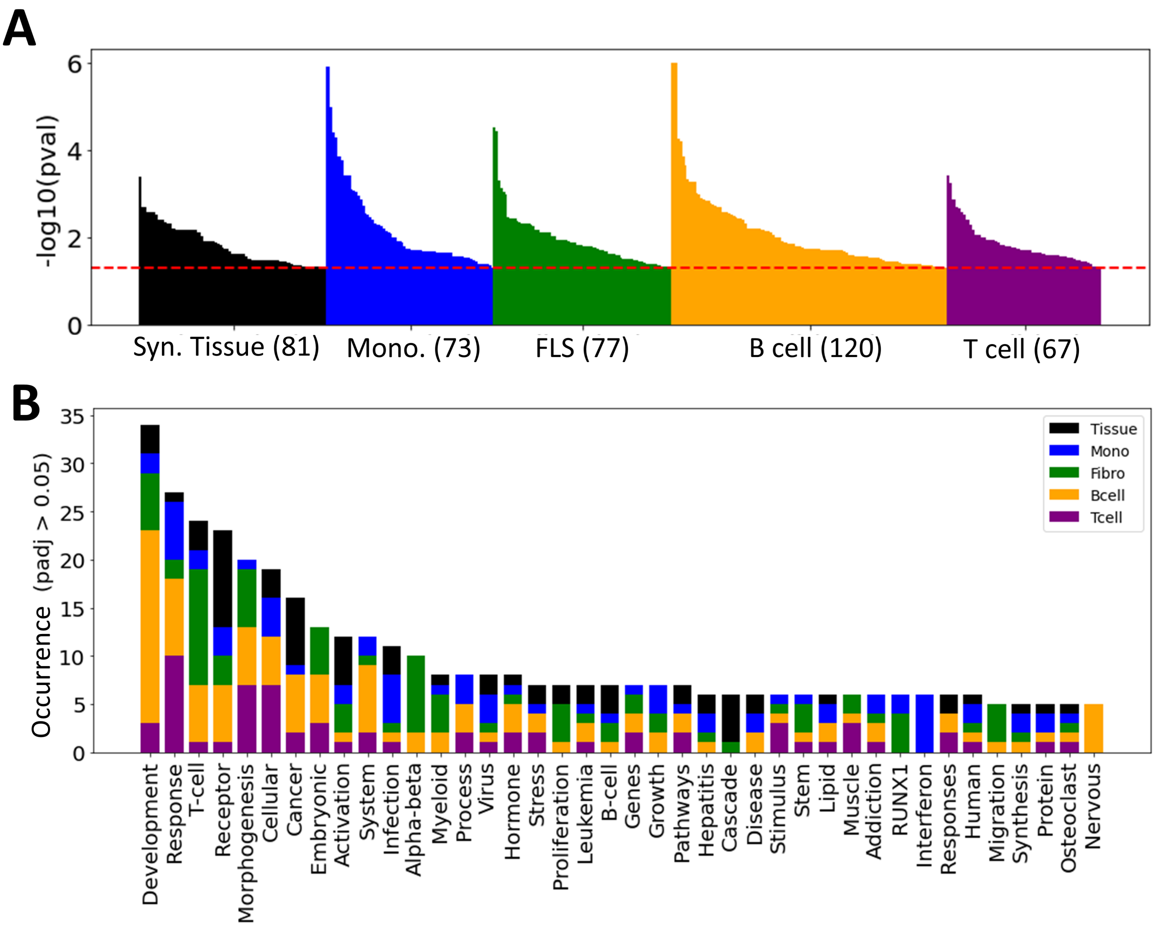

Figure S9: (A) Number of differentiated pathways. (B) Occurence of each word in all the pathway terms combined.

Figure S10: TF-TF co-regulation networks of each cell type analyzed in this article. Network showing the major TFs involved in FLS RA regulation, with nodes size proportional to both the degree and the TF regulatory score (t_{reg}). Nodes are colored according to their clusters.

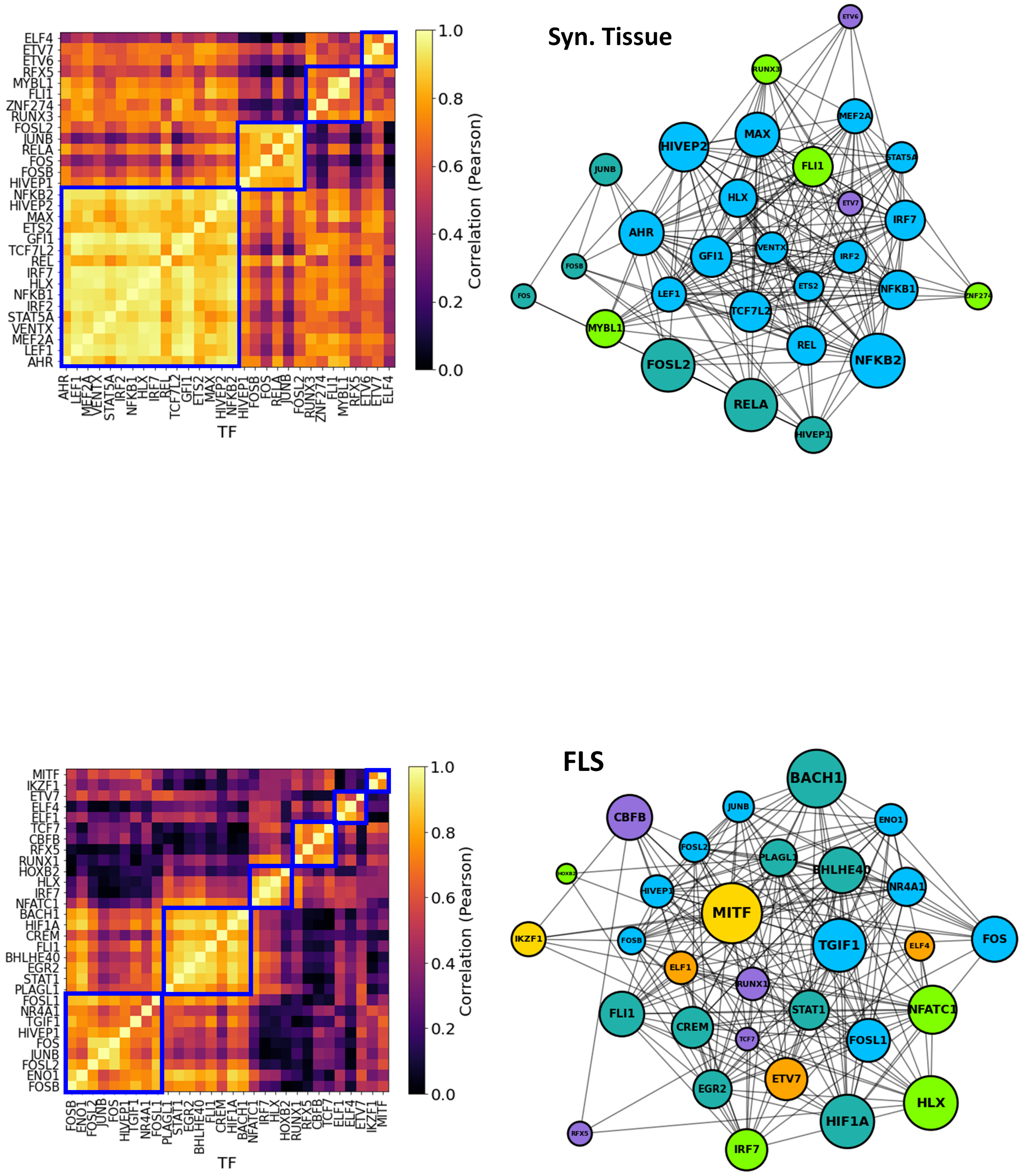

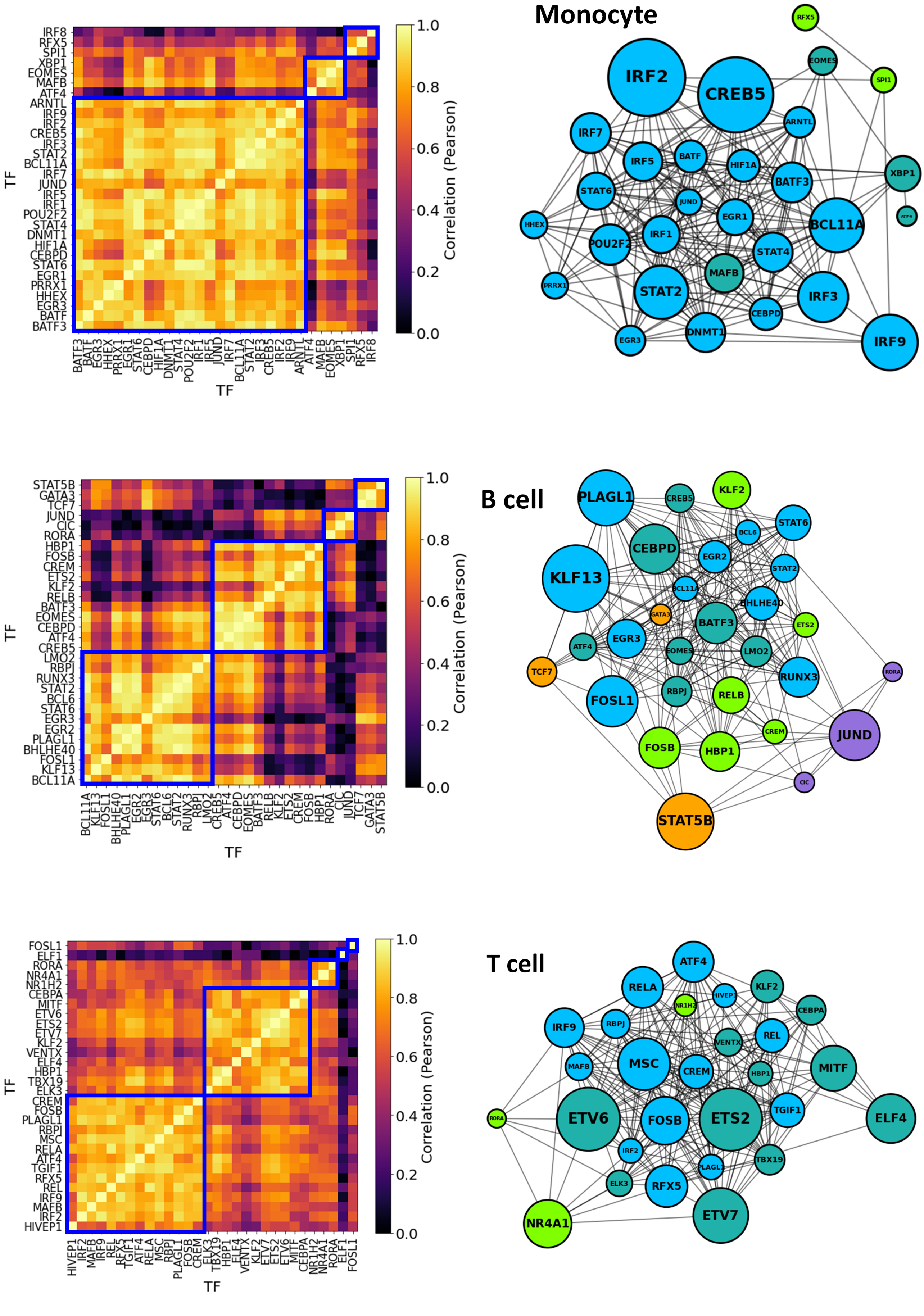

Monocyte
B cell
T cell
TF
TF
Correlation (Pearson)
1.0
0.8
0.6
0.4
0.2
0.0
